# A Monolithic, Thiol-Functionalized Au-Based Bio-CMOS Aptasensor for Rapid, Label-Free Detection of *Escherichia coli* O157:H7 in Patient-Derived and Hospital-Acquired Specimens

**DOI:** 10.3390/bioengineering13080858

**Published:** 2026-07-25

**Authors:** Zahra Nejad Shahrokh Abadi, M. H. Shahrokh Abadi, Reza Nejad Shahrokh Abadi

**Affiliations:** 1 Countess of Chester Hospital Foundation NHS Trust, Chester CH2 1UL, UK; 2 Electrical and Computer Engineering Faculty, Hakim Sabzevari University, Sabzevar 9617976487, Iran; 3 Living Materials Research Group, Hakim Sabzevari University, Sabzevar 9617976487, Iran; 4 Leipzig Center of Metabolism, Faculty of Medicine, Leipzig University, 04103 Leipzig, Germany; nshahrokhareza@gmail.com; 5 Department of Neurology, Max Planck Institute for Human Cognitive and Brain Sciences, 04103 Leipzig, Germany

**Keywords:** CMOS biosensor, aptasensor, point-of-care diagnostics, lipopolysaccharide, hospital-acquired infection, label-free detection

## Abstract

Rapid, point-of-care detection of *Escherichia coli* O157:H7 remains an unmet clinical need, as culture and molecular methods are slow and poorly suited to decentralized or emergency settings. A label-free, monolithic aptasensor biochip was fabricated in a standard 65 nm CMOS process, featuring three aptamer-functionalized gold sensing pads with matched reference pads for differential readout. A 37-mer DNA aptamer targeting the *E. coli* O157:H7 lipopolysaccharide was immobilized via thiol–gold self-assembled monolayer chemistry. Binding events were transduced into surface-potential shifts, amplified by an on-chip analog front-end (~100 V/V gain, 101.5 µW), and evaluated using calibration standards, patient specimens, and hospital environmental samples, with fluorescence microscopy for validation. The sensor achieved 47.42 mV/decade sensitivity across 1–10,000 CFU/mL, an IUPAC detection limit near 3.74 CFU/mL, and an empirical LOD of about 11 CFU/mL, with outputs tracking bacterial load and ~5.7% matrix-related deviation. Hospital samples were detectable to 28 CFU/mL. Because the patient-derived and hospital-acquired cohorts (*n* = 10 and *n* = 6, respectively) were assembled for pilot analytical and matrix-tolerance characterization rather than for diagnostic-accuracy determination, these results establish detectability and matrix robustness in real clinical and environmental specimens rather than clinical diagnostic sensitivity or specificity, which will require a larger, prospectively enrolled cohort in future work. Sensor kinetics followed Langmuir-type adsorption, saturating within 16–25 min for target pathogens versus slower responses for non-target strains. Selectivity tests against six bacterial species showed discrimination, with cross-reactivity decreasing from related *E. coli* pathotypes to Enterobacteriaceae to Gram-positive species. Inter-pad variability stayed below 1.5 mV, supporting this compact, low-power platform for scalable, enrichment-free point-of-care pathogen detection.

## 1. Introduction

### 1.1. Clinical Burden of E. coli O157:H7 in Healthcare Settings

Hospital-acquired infections (HAIs) caused by Shiga-toxin-producing *Escherichia coli* (STEC), particularly the O157:H7 serotype, represent a significant and growing public health challenge worldwide. Globally, STEC strains collectively account for an estimated 2.8 million acute illnesses annually, ranging from self-limiting gastroenteritis to life-threatening sequelae, including hemolytic uremic syndrome (HUS), hemorrhagic colitis, and acute renal failure [1,2,3]. In healthcare environments, *E. coli* O157:H7 can persist on surfaces, contaminate clinical specimens, and spread via patient contact pathways, contributing to nosocomial outbreaks and prolonged hospital stays [4,5,6]. Critically, even brief delays in pathogen identification can facilitate rapid bacterial dissemination and substantially worsen clinical outcomes, underscoring the urgency of timely diagnostic intervention [5,6,7,8,9].

Conventional clinical microbiology workflows depend on culture-based methods requiring up to 24–72 h of enrichment, selective plating, biochemical characterization, and serological confirmation before a definitive diagnosis is rendered. For a pathogen as clinically consequential as *E. coli* O157:H7 (where early antimicrobial management and infection control decisions depend directly on rapid confirmation), such delays are diagnostically unacceptable. Faster identification can shorten hospital stays, enable timely antimicrobial stewardship decisions, reduce secondary transmission within wards, and ultimately improve patient survival rates.

### 1.2. Existing Detection Methods: Capabilities and Limitations

A range of detection technologies has been developed for *E. coli* O157:H7, each presenting trade-offs between sensitivity, specificity, speed, cost, and operational complexity. Culture-based methods remain the gold standard but are inherently slow and require specialized infrastructure. Immunomagnetic separation and colony-counting assays improve throughput but do not eliminate the need for culture steps [10,11]. Molecular approaches, including PCR and quantitative PCR, offer high sensitivity and pathogen specificity but require thermocycling equipment and trained operators, and remain susceptible to PCR inhibition in complex clinical matrices [12,13]. Enzyme-linked immunosorbent assays (ELISAs) provide quantitative readout but involve multi-step protocols and batch processing unsuitable for real-time decision-making at the bedside [14]. Spectroscopic platforms including mid-infrared and Raman spectroscopy offer structural discrimination but demand costly instrumentation and non-trivial data interpretation [15,16].

Electrochemical biosensors and aptasensors have emerged as particularly promising alternatives, offering rapid, label-free, and miniaturizable detection of bacterial targets. Among these, aptamer-based electrochemical biosensors have demonstrated sensitivity in the 1–106 CFU/mL range for various pathogens, though many published platforms are validated exclusively in clean buffer environments [17,18,19,20] and have not been tested against real patient-derived or hospital-acquired specimens. The transition from buffer-only to clinical matrix performance represents one of the most significant and under-addressed gaps in the biosensor literature.

### 1.3. Aptamers as Biorecognition Elements for Pathogen Detection

Aptamers are short single-stranded nucleic acid molecules selected in vitro through the Systematic Evolution of Ligands by EXponential enrichment (SELEX) process. They fold into precise three-dimensional structures that bind target molecules with high affinity and specificity, often rivaling or exceeding antibody performance [21,22,23]. For bacterial detection, whole-cell SELEX has enabled the development of aptamers that recognize surface-exposed epitopes, including lipopolysaccharide (LPS), outer membrane proteins, and flagellar antigens, thus providing intact whole-cell recognition without requiring cell lysis or molecular extraction.

Compared to antibodies, aptamers offer several practical advantages for clinical biosensor integration: they are chemically synthesized with high batch-to-batch reproducibility, tolerate a wider range of storage conditions, are amenable to chemical modification (including thiol and amine functionalization for surface immobilization), exhibit lower production costs, and can, in principle, be regenerated through denaturation and refolding cycles. Their compatibility with gold and other electrode surfaces, through well-established thiol–gold self-assembled monolayer chemistry, makes them ideally suited for integration with CMOS-based biosensing platforms.

The 37-mer DNA aptamer employed in the present study was previously identified through cell-based SELEX for high-affinity recognition of the *E. coli* O157:H7 lipopolysaccharide, and its specificity and binding characteristics for this pathogen have been established in the literature.

### 1.4. CMOS-Integrated Biosensors: Current Landscape and Challenges

Complementary metal-oxide-semiconductor (CMOS) technology offers an attractive platform for biosensor miniaturization because it enables the monolithic co-integration of sensing electrodes, signal conditioning circuitry, and digital interfaces within a single chip, potentially reducing system complexity, footprint, and per-unit cost. CMOS-based biosensors, including ion-sensitive field-effect transistors (ISFETs) and extended-gate FET (EG-FET) configurations, have been applied to pH sensing, DNA hybridization detection, and, more recently, bacterial detection [24,25,26,27,28].

Recent lab-on-a-CMOS platforms have further extended monolithic on-chip integration to real-time bacterial growth kinetics monitoring [29], while self-calibrated capacitive readout front-ends have been proposed to reduce dependence on external reference circuitry in biosensor readout design [30]. Both directions are conceptually aligned with the reference-free, single-chip integration strategy adopted in the present work.

Nevertheless, several barriers limit the clinical translation of current CMOS biosensor platforms. Most reported devices have been validated exclusively in idealized buffer solutions, and few have been challenged with real patient-derived specimens or complex hospital-acquired matrices containing interfering proteins, salts, and non-target microorganisms. Additionally, many platforms rely on external reference electrodes, floating-gate architectures susceptible to electrostatic discharge, or off-chip signal amplification, all of which compromise robustness and portability. The absence of multi-channel designs further limits throughput and reproducibility assessment.

### 1.5. Knowledge Gap and Study Rationale

The clinical demand for rapid, bedside-deployable *E. coli* O157:H7 detection reveals a clear knowledge gap: existing biosensing platforms either lack real-world clinical validation, require external instrumentation that is incompatible with point-of-care deployment, rely on labeling or enrichment steps that add time and complexity, or have not demonstrated reproducible performance across multiple independently fabricated sensing channels. No previously reported CMOS aptasensor has been validated simultaneously in patient-derived specimens and hospital-acquired environmental samples while maintaining all detection, amplification, and reference circuitry on a single monolithic chip.

The present study addresses this gap by introducing and characterizing a monolithically integrated Bio-CMOS aptasensor that detects intact *E. coli* O157:H7 cells in real clinical matrices without enrichment, labeling, or external reference electrodes. The platform is validated across buffer calibration standards, residual patient specimens (PS series), and hospital-acquired environmental samples (HAS series), with fluorescence microscopy providing independent orthogonal validation. This work establishes the diagnostic analytical performance of the platform, identifies matrix effects quantitatively, and provides a practical foundation for future multi-pathogen, point-of-care diagnostic systems.

## 2. Materials and Methods

### 2.1. Reagents and Materials

Phosphate-buffered saline (PBS, 0.01 M, pH 7.2–7.4; Sigma-Aldrich, St. Louis, MO, USA, P2272) was used as the standard assay buffer throughout. 6-Mercapto-1-hexanol (MCH, 1–2 mM) for electrode passivation, TE buffer (10 mM Tris-HCl, pH 8.0, 1 mM EDTA) for aptamer reconstitution, and LB agar for plate counting were obtained from standard commercial suppliers. Commercially lyophilized *E. coli* (EC11303, *E. coli* Strain B; Sigma-Aldrich) served as the calibration organism. PDMS (polydimethylsiloxane) and curing agent were used for microwell fabrication.

### 2.2. Aptamer Sequence and Immobilization Chemistry

A 37-mer single-stranded DNA aptamer (5′-CCCTCCGGGGGGGTCATCGGGATACCTGGTAAGGATA-3′), previously identified through cell-based SELEX for high-affinity recognition of *E. coli* O157:H7 surface epitopes, particularly LPS moieties, was used as the biorecognition element. The aptamer was synthesized at a 200 nM scale, HPLC-purified (>95%), and obtained from BIONEER (Incheon, Republic of Korea), with a 5′-fluorescein (5-FAM) label for fluorescence-based immobilization verification and a 3′-thiol group enabling covalent attachment to gold sensing pads.

**Figure 1:**
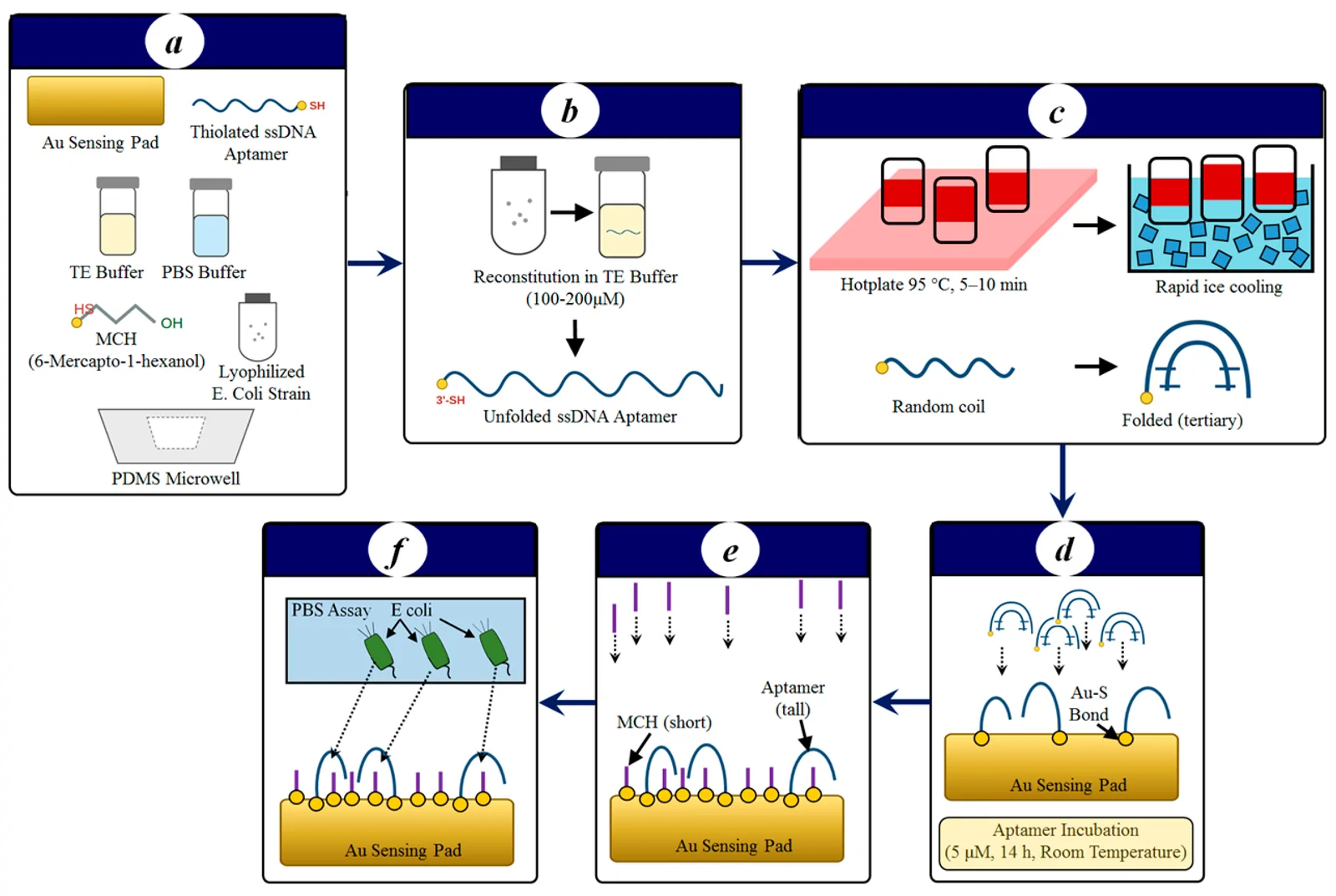
Workflow for thiol functionalization of Au sensing pads. (**a**) Main components for surface chemistry, (**b**) stock solution stored at ~20 °C before thermal annealing, (**c**) thermal annealing: heat disrupts secondary structure; rapid cooling locks in the functional 3D folds, (**d**) Au surface immobilization: self-assembled monolayer (SAM) formation. The thiolated 3′ terminus undergoes chemisorption onto the gold surface, forming a covalent Au–S (gold–thiolate) bond with loss of the thiol proton, anchoring single-stranded DNA (ssDNA) aptamers to the gold sensing pad; (**e**) surface 6-mercapto-1-hexanol (MCH) passivation (1–2 mM, 1–2 h). MCH backfills unoccupied Au sites, orienting aptamers upright, blocking non-specific binding, and reducing non-specific adsorption. (**f**) Functionalized Au pad, ready for specific recognition of *E. coli* O157:H7.

Prior to immobilization, lyophilized aptamer was reconstituted in TE buffer to 100–200 μM and thermally annealed (95 °C, 5–10 min; rapid ice-cooling) to promote correct tertiary folding. The thiol-modified aptamer was then incubated at 5 μM in immobilization buffer on the chip’s gold sensing pads for 14 h at room temperature, enabling covalent Au–S self-assembled monolayer (SAM) formation. Unoccupied gold sites were subsequently passivated with 1–2 mM MCH for 1–2 h to minimize non-specific adsorption and maintain aptamer accessibility. Figure 1 summarizes the functionalizing sensing pads’ workflow.

### 2.3. Bio-CMOS Sensor Fabrication and Platform Description

The Bio-CMOS biochip was fabricated using a standard 65 nm TSMC CMOS process. The chip (1.5 mm × 0.7 mm) incorporates three independent sensing blocks; each sensing block comprises an aptamer-functionalized gold sensing pad (69 µm × 57 µm) paired with a geometrically identical reference pad that is also functionalized with the same aptamer/MCH surface chemistry. During measurements, the reference pad receives PBS to provide differential baseline compensation through the Out2 readout. The sensing architecture employs an extended-gate field-effect transistor (EG-FET) configuration in which the sensing pad is physically separated from the transistor channel, enhancing robustness against liquid-induced degradation. An on-chip analog front-end consisting of two cascaded amplifier stages (total gain of ~100 V/V, bandwidth of 50 kHz, total static power of 101.5 μW at 1 V supply) conditions the differential signal between sensing and reference pads for acquisition.

**Figure 2:**
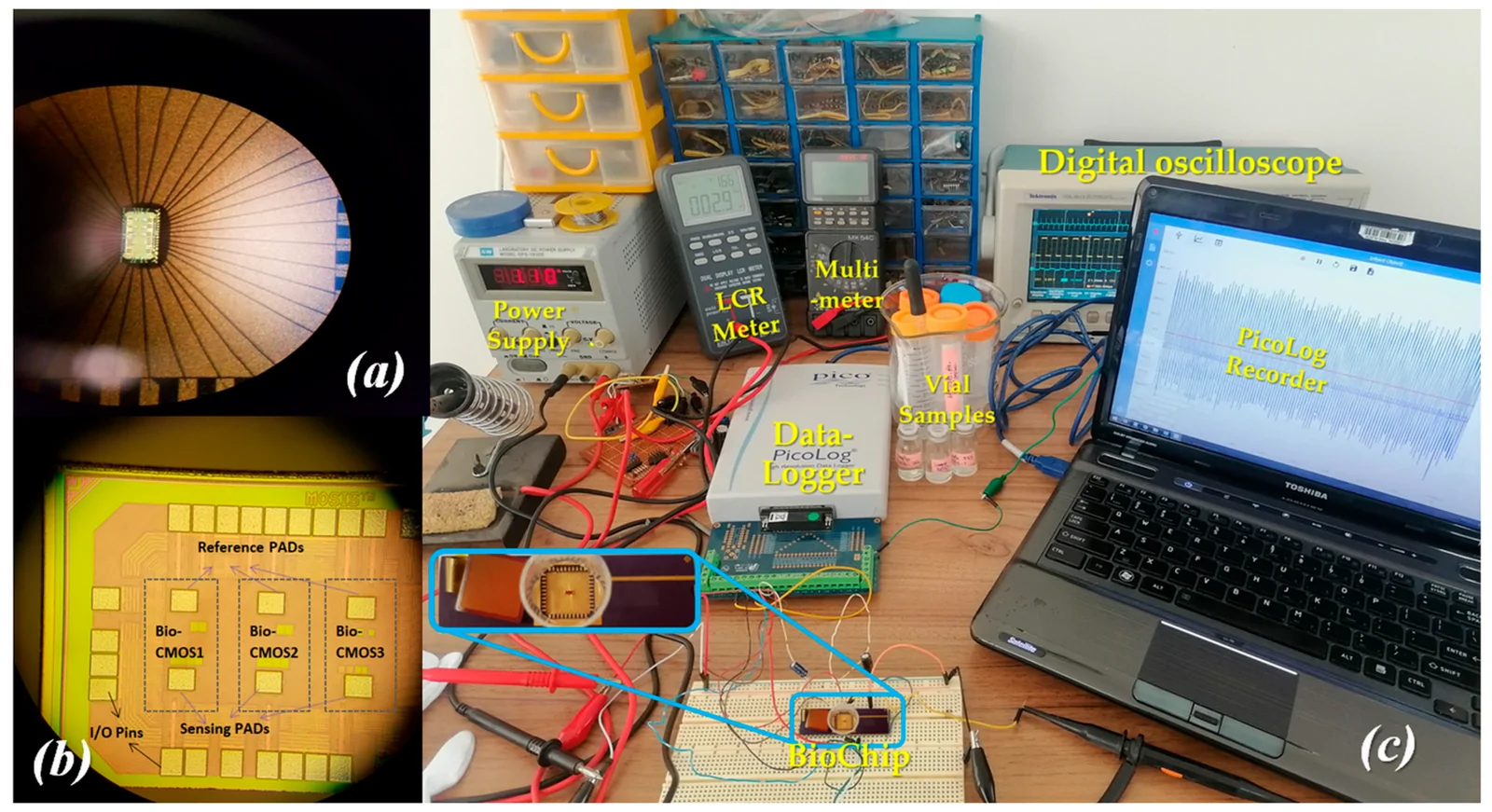
(**a**) Fabricated 65 nm CMOS biochip die (1.5 mm × 0.7 mm) showing the integrated analog front-end circuitry and the dedicated gold sensing region (69 µm × 57 µm) designed for aptamer functionalization and label-free bacterial detection, (**b**) biochip wire-bonded and packed in a DIP-40 carrier and post-processed with PDMS microwells aligned over the sensing and reference pads, enabling controlled sample delivery, incubation, and real-time electrical interrogation of clinical and calibration specimens, and (**c**) complete experimental setup for electrical and biosensing characterization of the CMOS biochip.

PDMS microwells were fabricated and aligned over the sensing and reference pads to enable controlled sample delivery and incubation. The packaged chip (DIP-40 carrier) was mounted on a custom breadboard for benchtop characterization. Output voltages (Out2) from all three sensing channels were acquired simultaneously via a PicoLog data logger at 6 s intervals. Circuit-level details of the biasing, amplifier topology, and reference generation are provided in the Supplementary Materials; only specifications directly relevant to biosensor performance are reported here. Figure 2 illustrates the experimental setup for electrical and biosensing characterization.

### 2.4. Clinical Sample Collection and Ethical Considerations

This pilot clinical feasibility evaluation used discarded, fully anonymized residual clinical specimens provided by a partner diagnostic laboratory after the completion of routine PCR and fluorescence confirmation testing, with the explicit goal of assessing detectability and matrix tolerance rather than establishing diagnostic accuracy. No identifiable patient information was collected, accessed, or used at any stage, and no direct patient contact occurred. The Hakim Sabzevari University Ethics Committee (Sabzevar, Iran) reviewed the procedure and determined that formal ethical approval was not required for this minimal-risk research involving de-identified, discarded specimens. A waiver of ethical approval was granted accordingly.

Residual specimens originated from two categories: (1) diagnostic specimens from patients with confirmed *E. coli* O157:H7 infections (PS series) and (2) environmental swabs from hospital-acquired contamination sites (HAS series). All samples were fully anonymized before transfer to the research team.

### 2.5. Preparation of Bacterial Suspensions

Calibration standards were prepared from lyophilized EC11303 (Sigma-Aldrich) reconstituted in 0.01 M sterile PBS according to the manufacturer’s instructions. Serial dilutions yielded four calibration concentrations, 10 CFU/mL (TS-E1), 10^2^ CFU/mL (TS-E2), 10^3^ CFU/mL (TS-E3), and 10^4^ CFU/mL (TS-E4), each verified in triplicate by plate counting on LB agar. Clinical specimens were processed immediately upon receipt (0–2 °C transport, within 2–4 h): low-speed centrifugation (3000–5000 rpm, 5–10 min) removed particulate debris; supernatants were diluted in PBS (pH 7.2) to standardize ionic strength and reduce matrix-induced variability. This dilution step served two purposes: removal of residual particulate debris prior to microwell loading, and standardization of ionic strength across specimen types, which, as discussed in Section 4, also increases the Debye length relative to undiluted physiological fluid (≈150 mM), a recognized strategy for preserving field-effect sensitivity in aptamer- and antibody-based FET biosensors. Aliquots were prepared for direct native-matrix testing, spiked controls with known *E. coli* concentrations, and negative PBS controls.

### 2.6. Biosensing Measurement Procedure

Sensing pad wells were filled with bacterial suspension or clinical/environmental samples; reference pad wells received equal volumes of PBS for differential baseline subtraction. The biochip was incubated at room temperature for 40–60 min to allow aptamer–bacteria binding equilibration, after which the supernatant was removed and wells were gently rinsed with PBS. A post-wash rest period of 15–20 min was applied before final readout to ensure signal stabilization. Out2 voltages, representing steady-state differential surface-potential shifts, were recorded across all three sensing pads. Each sample, including calibration standards, patient-derived and hospital-acquired specimens, and the non-target bacterial species (described in Section 2.7), was tested using this identical protocol, with at least two independent loading cycles per sensing pad; reported values are means ± *SD* across pads and replicates. Fluorescence microscopy was performed on representative TS, PS and HAS specimens using a 5′-FAM-labeled aptamer preparation to independently confirm the presence and density of bound *E. coli* O157:H7 at the sensing surface, providing orthogonal validation of the electrical measurements consistent with the fluorescence findings reported in Section 3.

### 2.7. Analytical Performance Evaluation

The limit of detection (LOD) was calculated using the standard IUPAC criterion, LOD = 3*σ*_blank_/*S*, where *σ*_blank_ is the standard deviation of Out2 under analyte-free conditions (PBS, *n* ≥ 5) and *S* is the slope of the calibration curve (Out2 vs. log_10_ CFU/mL). Sensitivity was defined as the slope of the calibration function in mV/decade. Matrix effects were quantified by comparing Out2 responses at matched bacterial loads between buffer calibration standards and patient-derived samples. Inter-pad reproducibility was assessed from the standard deviation across the three parallel sensing channels for each sample. The calibration function was fitted as an unconstrained linear regression, Out2 = a + b × log_10_[CFU/mL], rather than a zero-intercept model, because (i) log_10_[CFU/mL] has no physically defined zero-concentration point on this axis, and (ii) blank (analyte-free) measurements showed a small but reproducible non-zero baseline response, consistent with standard IUPAC/analytical-chemistry calibration practice of not constraining the intercept absent of independent physical justification.

To account for the sensor’s saturating response at the extremes of the tested concentration range, the calibration function was additionally fitted to a four-parameter logistic model, and the limit of detection was re-estimated from the local slope of this nonlinear function evaluated near the blank, following the calibration-uncertainty approach described for label-free biosensors by [31].

## 3. Results

### 3.1. Biochip Characterization and Platform Stability

The Bio-CMOS chip was fabricated in a 65 nm TSMC CMOS process, yielding a die footprint of 1.5 mm × 0.7 mm with a dedicated gold sensing region of 69 μm × 57 μm per channel. The platform operates at a 1 V supply with a total static power consumption of 101.5 μW (Table 1), confirming suitability for battery-powered portable diagnostic applications. The on-chip analog front-end provides approximately 40 dB gain (100 V/V) with a flat frequency response up to 50 kHz and an input-referred noise of 16.6 μVrms integrated over the detection bandwidth. These electrical properties establish a minimum resolvable surface-potential change of approximately 50 μV at the sensing pad, sufficient to detect the mV-range surface-potential perturbations generated by aptamer–bacteria binding.

**Figure 3:**
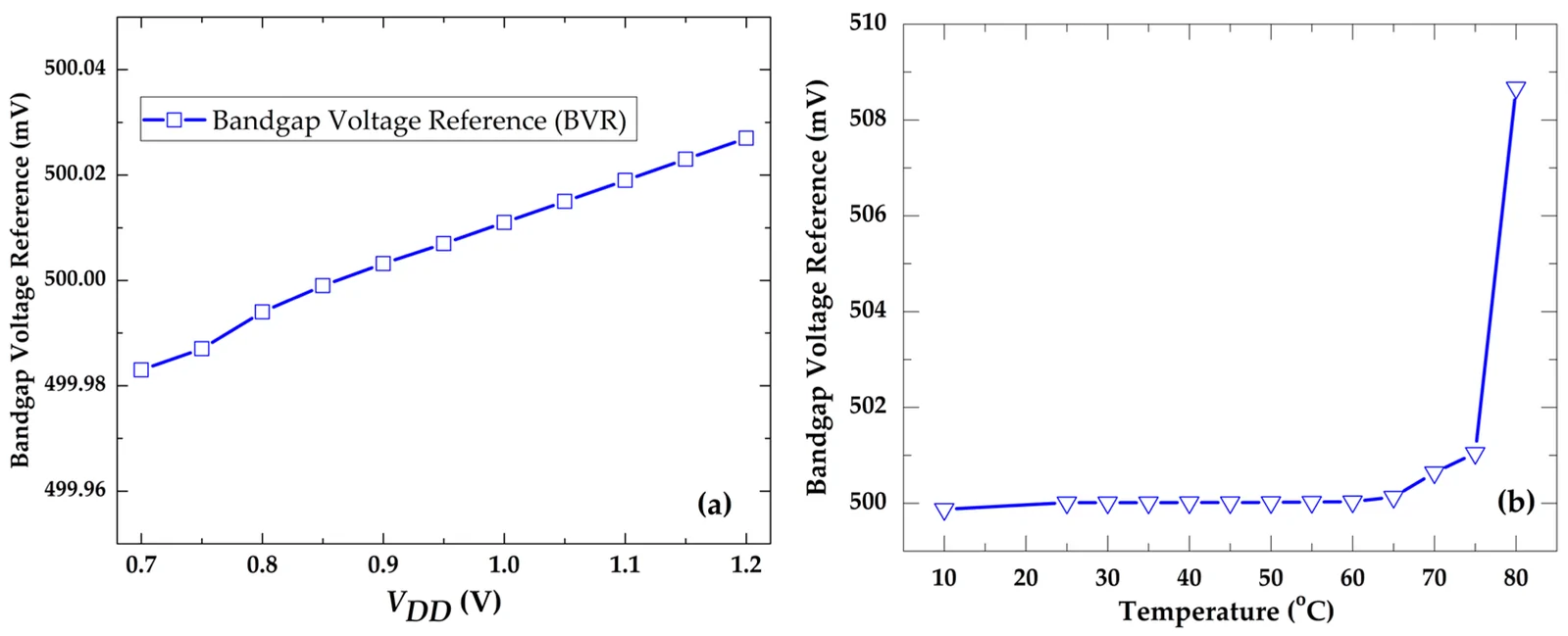
(**a**) Bandgap voltage reference output stability across supply variations (0.7–1.2 V), achieving ≈ 0.088 mV/V fluctuation; (**b**) the BVR voltage temperature dependence in the range of 10 °C to 80 °C, showing a maximum variation of 8.47 mV.

Following aptamer immobilization and MCH passivation, baseline Out2 voltages remained within a narrow 0.7–1.2 mV window over a 180 min observation period with no measurable drift, confirming that the aptamer–MCH monolayer forms a chemically stable and electrostatically quiescent interface. This absence of baseline drift is an essential prerequisite for reliably attributing subsequent signal changes to specific bacterial binding events rather than surface instability or circuit artifacts. The on-chip reference circuit exhibited supply stability of 0.088 mV/V across 0.7–1.2 V and a maximum thermal shift of 8.47 mV between 10 and 80 °C (Figure 3), providing a stable reference throughout clinical sample measurements conducted at ambient temperatures. The modest additional deviation observed between 75 °C and 80 °C reflects residual second-order curvature in the bandgap reference, arising from the non-ideal (super-linear) temperature dependence of the complementary-to-absolute-temperature (CTAT) voltage term and increased subthreshold sensitivity of the biasing transistors at an elevated temperature, a well-documented characteristic of first-order-compensated bandgap topologies. This regime lies well above the ambient temperatures (20–25 °C) at which all sensing measurements in this study were performed and therefore does not affect reported biosensor performance. The system exhibits a flat gain of approximately 40 dB from low frequencies up to 50 kHz, with no observable peaking, indicating stable compensation and adequate phase margin (Supplementary Material Figure S1), with a complete architecture shown in Supplementary Material Figure S2. Furthermore, the design detail of bandgap voltage reference is illustrated in Supplementary Material Figures S3 and S4. The details of component features for BVR and current biasing blocks are given in Supplementary Material Table S1 and Supplementary Material Table S2, respectively.

### 3.2. Surface Modification and Verification of Aptamer Functionalization

**Figure 4:**
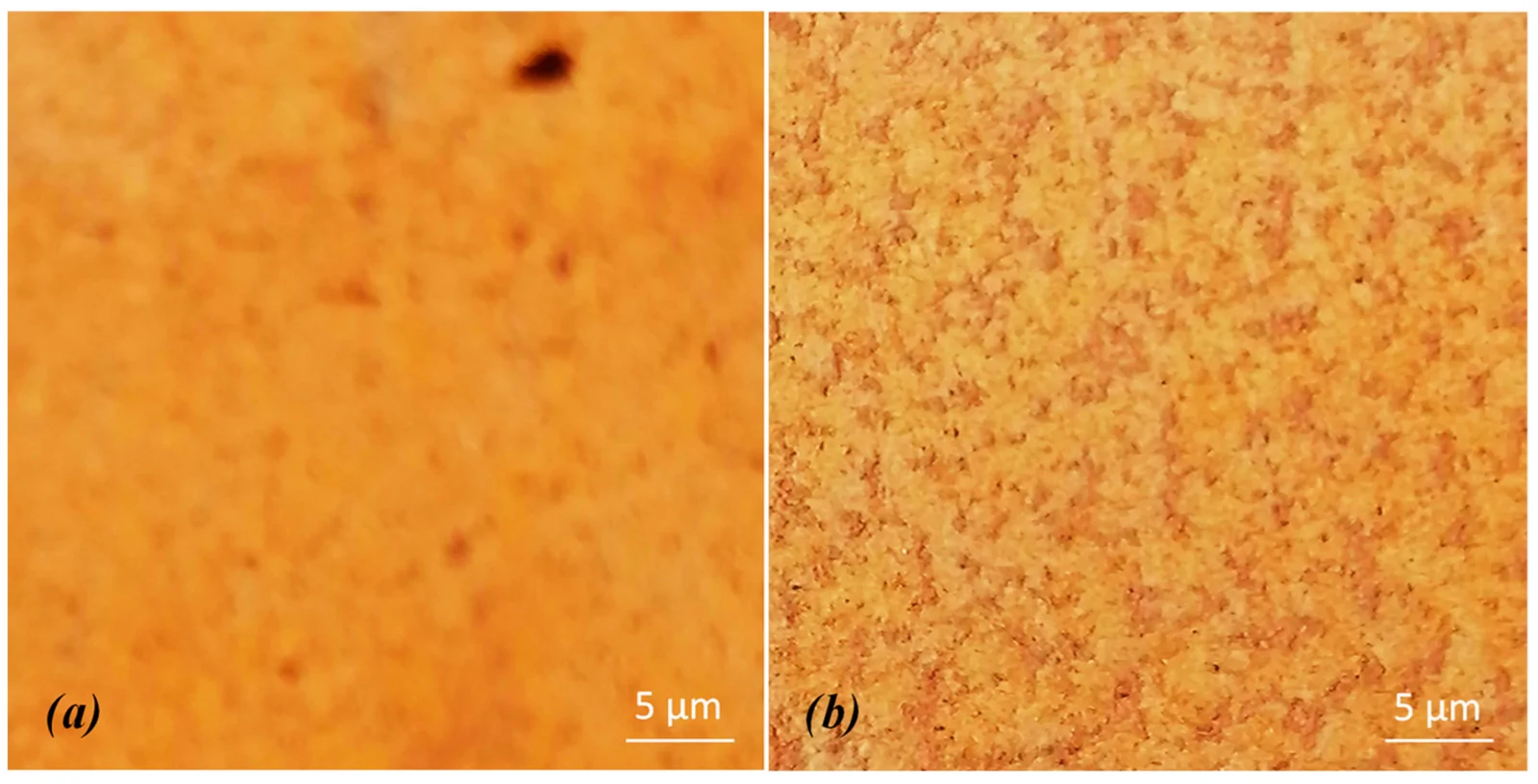
Optical microscopy images of the Au pad surface: (**a**) before and (**b**) after cleaning procedures.

Prior to functionalization and testing, the sensing pads were activated to ensure surface sensitivity. The Au pads were cleaned in acetone and isopropanol and rinsed with distilled water. Optical microscopy revealed particulate contamination on the as-received Au pads prior to cleaning. Figure 4 illustrates one of the Au pads (Out2) before and after modification, promoting homogeneity on the surface of the Au pads.

**Figure 5:**
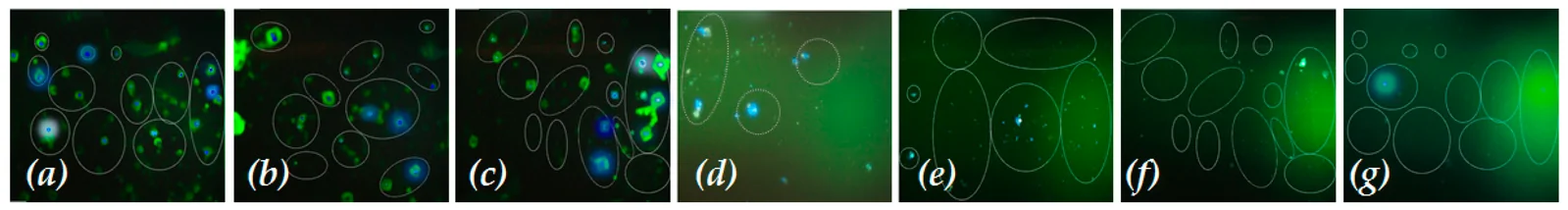
Fluorescence microscopy validation of *E. coli* O157:H7 detection in patient-derived (PS) and hospital-acquired (HAS) samples, accompanied by quantitative electrical readout from the Bio-CMOS aptasensor. Panels (**a**–**g**) show representative fluorescence micrographs of PS-4.27E3, PS-2.5E3, PS-1.07E3, HAS-87, HAS-73, HAS-31, and PS-11, respectively. White circles denote regions of interest (ROIs) where E. coli binding was expected/targeted for detection. Blue fluorescent puncta within these ROIs indicate successful aptamer–E. coli binding events, with darker and more intense blue signal corresponding to higher bacterial concentrations. The diffuse green fluorescence observed across all panels represents background, non-specific autofluorescence from the sample matrix and does not indicate specific detection.

Fluorescence microscopy was used to independently confirm successful aptamer immobilization and *E. coli* O157:H7 capture on the sensing surface (Figure 5). PS samples (PS series) with confirmed bacterial contamination produced intense green/blue fluorescence localized within the sensing region, consistent with dense aptamer–bacteria complex formation (Figure 5a–c). HAS samples with low bacterial loads correspondingly displayed weak, near-background fluorescence. The strong spatial concordance between the fluorescence signal and the sensing region, combined with the absence of comparable fluorescence on reference pads, confirms that the observed signal originates from a specific aptamer–LPS interaction rather than non-specific adsorption.

### 3.3. Nonlinear Recalibration, Model Comparison, Sensitivity, and Limit of Detection

Because the calibration response is generated by a finite population of surface-immobilized aptamer binding sites, a saturating (sigmoidal) dose–response relationship, rather than a strictly linear one, is the mechanistically expected functional form. To evaluate this quantitatively, the calibration data (Table 2) were additionally fit to a four-parameter logistic (4PL) model, *Out2* = *d* + (*a* − *d*)/[1 + (*C*/*c*)*^b^*], where *a* and *d* denote the lower and upper signal asymptotes, *c* is the inflection-point concentration, and *b* is the Hill-type slope factor, using nonlinear least-squares regression on the full replicate-level dataset. The 4PL model substantially improved the goodness of fit relative to the original linear model (R2 improving from 0.942 to >0.999), and its residuals showed no systematic structure, in contrast to the linear model’s residuals, which were systematically bowed, under-predicting the response at the lowest and highest tested concentrations. These results confirm that the linear model, while adequate as a local working approximation within the mid-range of the dynamic range, does not fully capture the sensor’s true saturating response, consistent with the reviewer’s observation, and that the 4PL model provides a more mechanistically faithful and statistically supported description of the calibration function across the full tested range. The limit of detection was correspondingly re-estimated from the local slope of the 4PL calibration function evaluated near the blank (tangent at *C* → 0), following the calibration-uncertainty framework of Lavín et al. [31], rather than a single global linear slope; this refined LOD estimate is reported alongside the original IUPAC linear-slope estimate for transparency. We note that, with five non-zero calibration levels, the lower asymptote and Hill-slope parameters of the 4PL fit carry wider uncertainty than the inflection point and upper asymptote; additional calibration points, particularly beyond the current upper concentration limit, would further constrain these parameters in future work.

**Figure 6:**
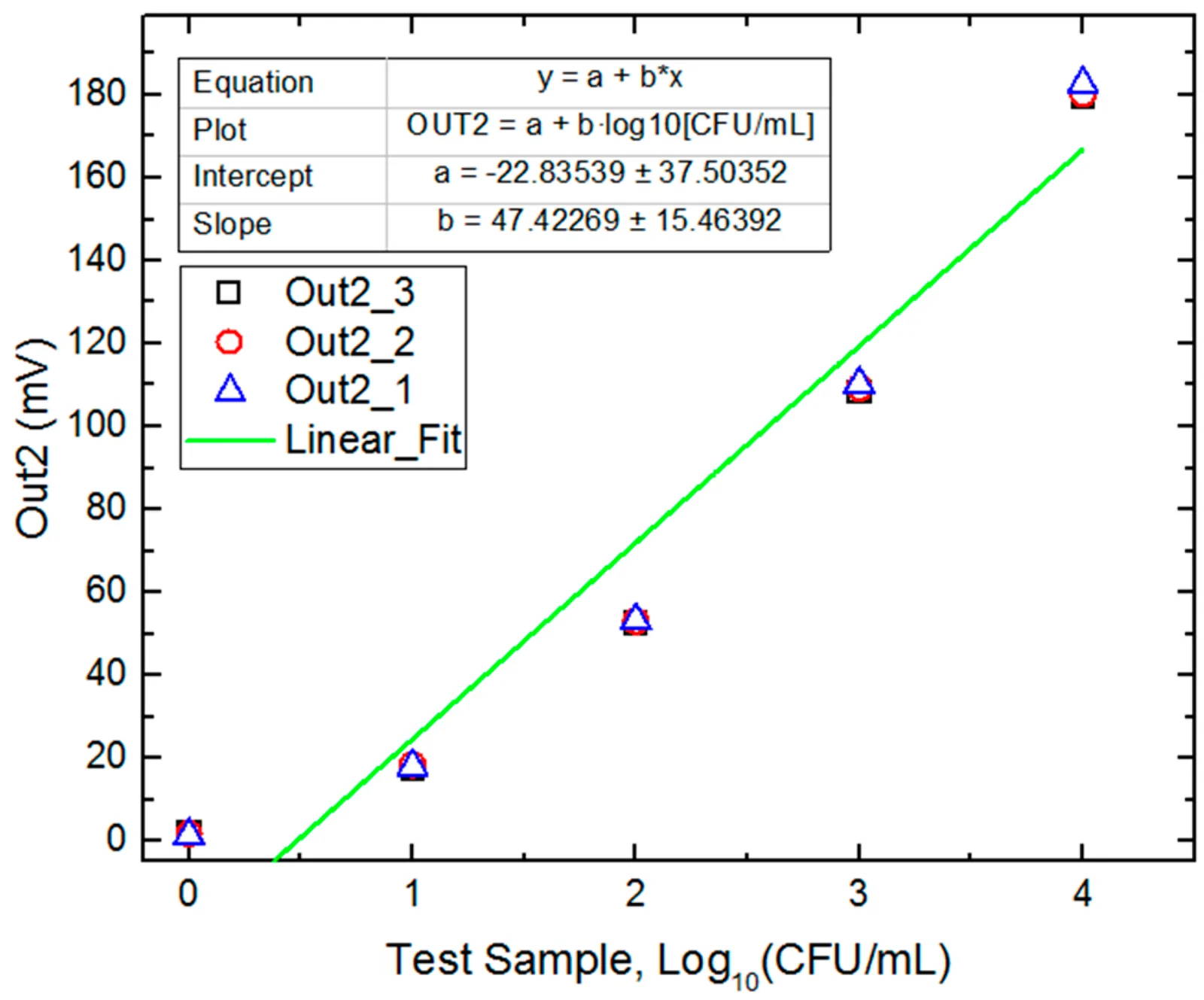
Responses of the Out2 sensing pad to different concentrations of test samples (TSs) are plotted on a semi-log scale (Out2 vs. log_10_ CFU/mL), together with a linear least-squares fit (Out2 = *a* + *b* × log_10_[CFU/mL]; *b* = 47.42 ± 15.46 mV/decade, *a* = −22.84 ± 37.50 mV), which is used to estimate sensitivity and limit of detection. The inset shows mean ± *SD* (*n* ≥ 3) responses of Out2_2 (middle sensing pad) at each concentration.

Calibration experiments using TS-E0 (1 CFU/mL), TS-E1 (10 CFU/mL), TS-E2 (100 CFU/mL), TS-E3 (1000 CFU/mL), and TS-E4 (10^4^ CFU/mL) standards produced Out2 voltages of ~1 mV to ~183 mV, demonstrating a clear monotonic, concentration-dependent response across four orders of magnitude (Table 2; summarized graphically in Supplementary Material Figure S5). The calibration slope was 47.42 mV/decade (sensitivity ≈ 47 mV/decade), and baseline noise (an average *σ*_blank_ ≈ 0.986 mV from aptamer + MCH functionalized pads in PBS) yielded an IUPAC analytical LOD of ~3.74 CFU/mL (Figure 6). Because the calibration curve deviates modestly from strict linearity at the extremes of the tested range (Section 3.4), this linear fit is best interpreted as a practical working approximation over the assay’s dynamic range rather than an exact mechanistic description of sensor response; the associated parameter uncertainty (slope 47.42 ± 15.46 mV/decade) should be borne in mind when interpreting the extrapolated LOD value.

### 3.4. Dynamic Range, Linearity, and Reproducibility

The sensor maintained a proportional response over a four-order-of-magnitude concentration range (approximately 10–10,000 CFU/mL, covering the range from the empirical LOD to the upper calibration limit). Slight deviation from strict linearity at the highest concentrations (10^4^ CFU/mL) is consistent with partial surface saturation of aptamer binding sites, a phenomenon well documented in surface-based biosensors. Within the dynamic range, the platform supports semi-quantitative estimation of bacterial load directly from measured Out2 voltage, which may be valuable for clinical triage and infection severity assessment.

Three independent sensing pads fabricated on the same chip produced closely aligned Out2 profiles for each sample (Table 2), with inter-pad standard deviations consistently below 1.5 mV across calibration standards. This high inter-pad agreement confirms that the aptamer immobilization procedure and CMOS fabrication process yield reproducibly uniform sensing interfaces, a critical requirement for reliable clinical diagnostics.

### 3.5. Patient-Derived and Hospital-Acquired Environmental Samples

**Figure 7:**
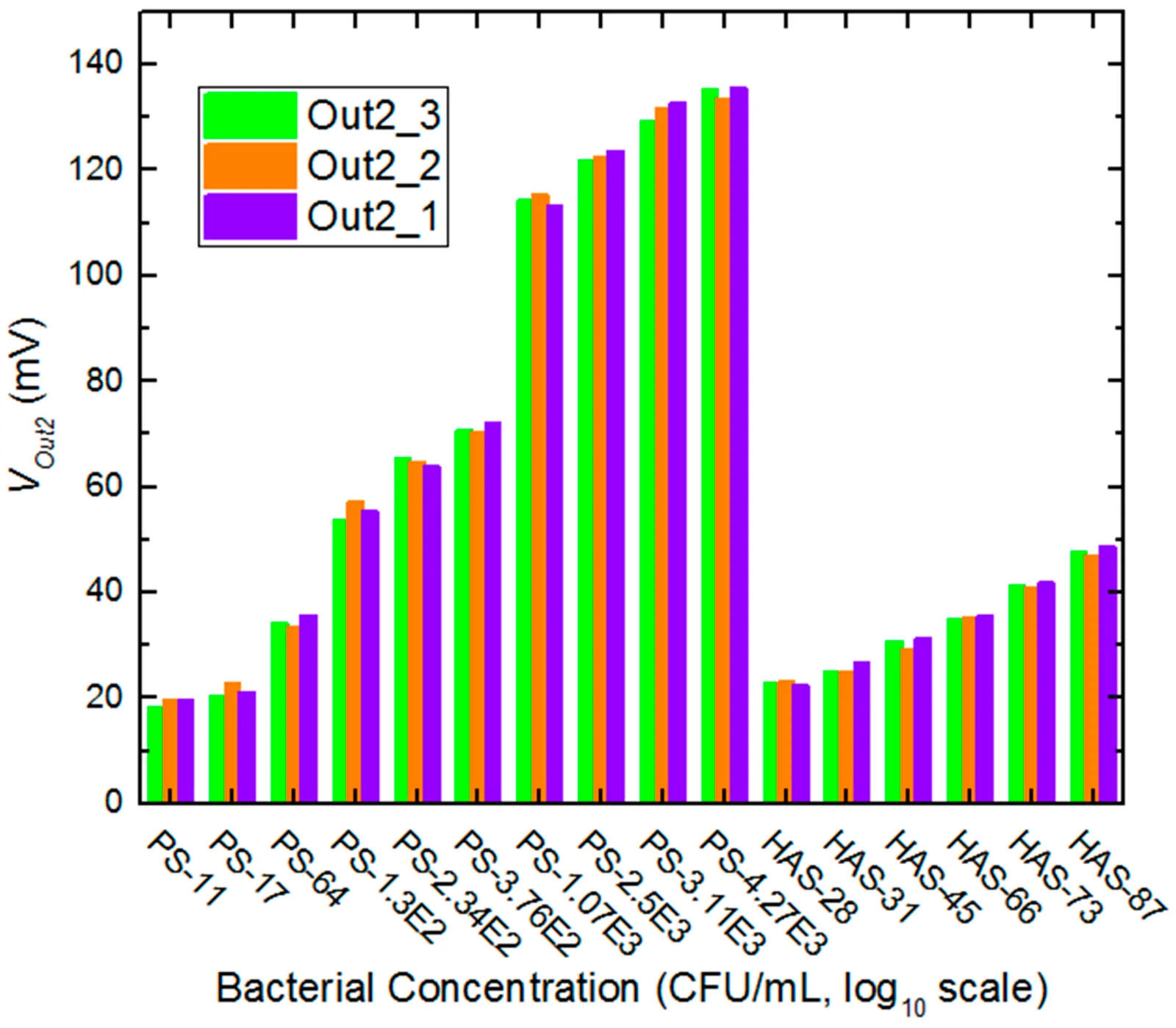
Correlation between bacterial concentration and Out2 output voltage across three independent sensing pads for patient-derived samples (PS series) and hospital-acquired samples (HAS series), spanning the PS and HAS series concentration range, ~11–4270 CFU/mL. The monotonic increase in signal amplitude demonstrates the linearity and reproducibility of the Bio-CMOS aptamer sensor over four orders of magnitude; the overlaid TS series calibration line (Figure 6) is shown for reference.

Ten patient-derived samples (PS series) spanning concentrations from 11 to 4270 CFU/mL were analyzed. The sample with 11 bacteria (PS-11) produced Out2 responses distinguishable from the aptamer–MCH baseline (~18–20 mV range), and was identified as the lowest clinically detectable specimen, near the detection threshold TS-E1, which can be considered an empirical LOD ≈ 11 CFU/mL. Sample PS-17 (17 CFU/mL) produced voltages of ~20–23 mV, while PS-1.3E2 through PS-4.27E3 showed monotonically increasing responses consistent with the buffer calibration trend. A direct comparison between the calibration standard TS-E2 (100 CFU/mL in PBS, mean Out2 ≈ 53 mV) and the patient sample PS-1.3E2 (~130 CFU/mL in clinical matrix, mean Out2 ≈ 56 mV) indicates a relative matrix-induced deviation of approximately ~5.7%, suggesting that the complex protein and ionic environment of patient specimens introduces only modest signal perturbation while preserving the fundamental detection capability. The empirical LOD, defined as the lowest bacterial concentration producing a reliably positive signal across all three sensing pads in clinical matrices, was ≈11 CFU/mL (PS-11). The slight sub-linearity observed at the low end of the calibration curve (Figure 7) is consistent with diffusion-limited binding kinetics within the fixed incubation window (Section 3.7), which would be expected to disproportionately affect the lowest-concentration samples.

Hospital-acquired specimens (HAS series, *n* = 6) representing environmental swabs from contamination-associated sites produced Out2 voltages ranging from ~20 mV (HAS-28) to ~49 mV (HAS-87). HAS-45 (≈45 CFU/mL) yielded Out2 values of ~30–31 mV across all three pads, consistent with the extrapolated calibration curve between TS-E1 and TS-E2, confirming that hospital matrix composition does not substantially suppress detection compared to test samples. HAS-28 and HAS-31 produced measurable signals near the threshold, correctly indicating above-LOD bacterial loads in those specimens. These results support the platform’s utility for environmental surveillance and infection control screening in healthcare settings.

The concordance between fluorescence microscopy and electrical Out2 responses across both specimen categories provides strong orthogonal evidence that the measured signals originate specifically from *E. coli* O157:H7 captured by the aptamer layer, rather than from non-specific adsorption or matrix artifacts.

### 3.6. Selectivity Experiments

Analytical selectivity of the Bio-CMOS aptasensor was evaluated using both closely related and clinically relevant non-target bacterial species. Commercially available lyophilized cultures of *Escherichia coli* K-12 MG1655, enterotoxigenic *E. coli* (ETEC H10407), Salmonella enterica serovar Typhimurium ATCC 14028, *Klebsiella pneumoniae* ATCC 13883, *Staphylococcus aureus* ATCC 25923, and *Enterococcus faecalis* ATCC 29212 were reconstituted and prepared under the same conditions used for *E. coli* O157:H7 calibration standards. Bacterial suspensions were adjusted to 10^2^ and 10^4^ CFU/mL in PBS and introduced individually into the PDMS microwells. Sensor responses were recorded using the same acquisition protocol employed for calibration experiments. Relative cross-reactivity was quantified by normalizing the output voltage generated by each non-target species to the response obtained from *E. coli* O157:H7 at the same concentration. Table 3 represents the cross-reactivity of enterobacteriaceae samples at 10^2^ and 10^4^ CFU/mL concentrations compared with TS-E2 and TS-E4.

The observed cross-reactivity gradient is mechanistically consistent with the aptamer’s lipopolysaccharide-directed recognition mechanism. Lipopolysaccharide is a structural feature unique to the Gram-negative outer membrane; the two Gram-positive organisms tested (*S. aureus*, *E. faecalis*), which lack LPS entirely, accordingly produced near-baseline responses (2–7% relative signal), consistent with the absence of the aptamer’s target epitope rather than with a matrix or non-specific-adsorption artifact, a distinction supported by the differential (sensing-minus-reference-pad) readout and MCH surface passivation used throughout this study. Among the Gram-negative organisms tested, cross-reactivity decreased with decreasing O-antigen/LPS structural homology to *E. coli* O157:H7, from other *E. coli* pathotypes (12–22%) to Salmonella Typhimurium and *Klebsiella pneumoniae* (5–15%), consistent with the aptamer engaging a conserved but not genus-universal LPS epitope.

### 3.7. Adsorption Kinetics and Response-Time Characterization of the Aptasensor

**Figure 8:**
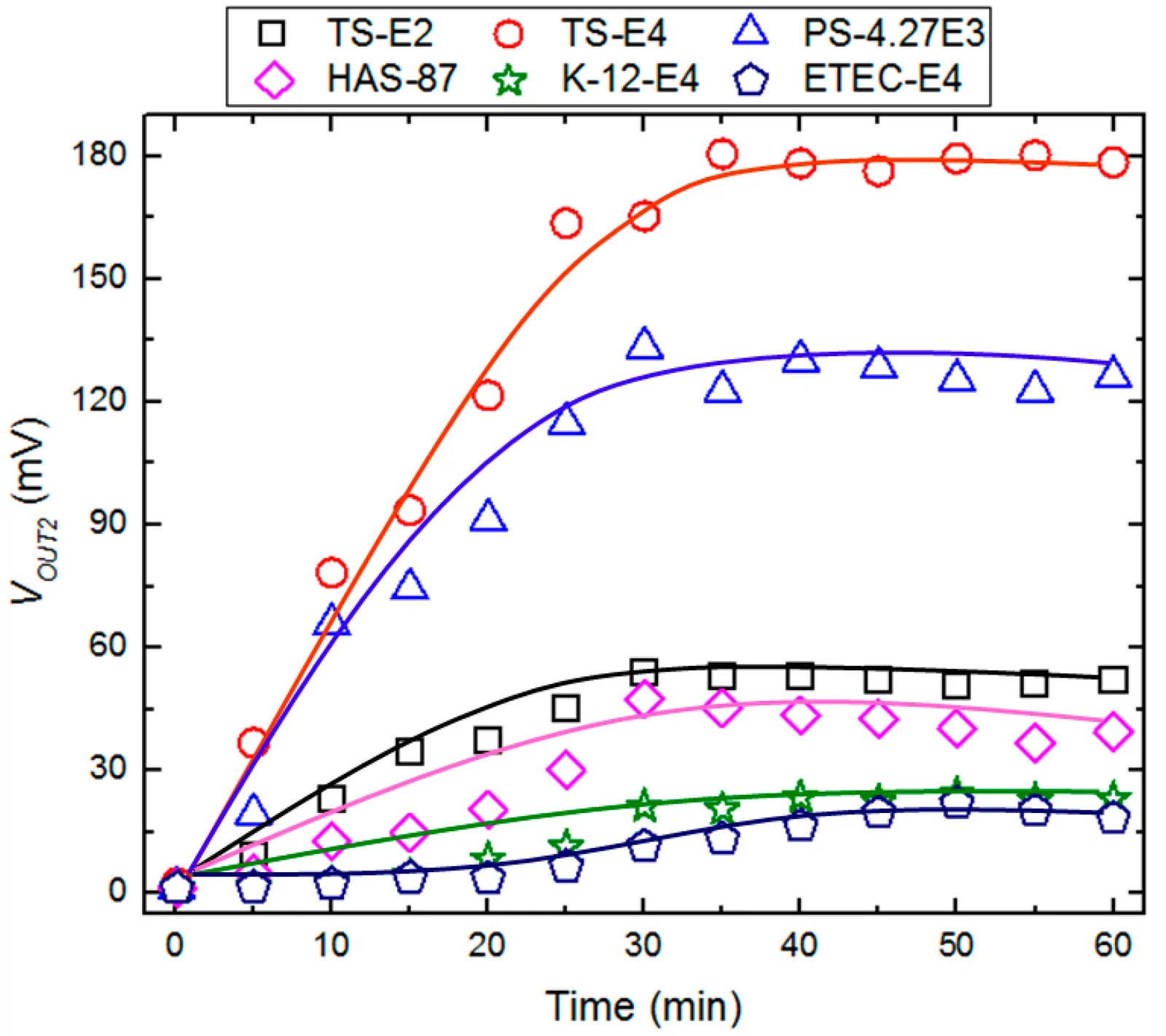
Temporal response of the sensing pads (*V_Out2_*) to six representative analytes over a 60 min incubation period. Symbols represent measured data points (Supplementary Table S3); solid lines represent nonlinear least-squares fits to the single-exponential (pseudo-first-order Langmuir) adsorption model, *V_Out2_*(*t*) = *V_max_*(1 − e^−*t/τ*^). Fitted parameters for each analyte are reported in Table 4.

The temporal response of the sensing pad, *V_Out2_*(*t*), to six representative analytes (TS-E2, TS-E4, PS-4.27E3, HAS-87, K12-E4, and ETEC-E4) was monitored continuously over a 60 min incubation period to characterize the approach to steady state (raw data provided in Supplementary Material Table S3). For all analytes tested, *V_OUT2_*(*t*) increased monotonically before plateauing, and the resulting response curves (Figure 8) closely followed the exponential rise-to-equilibrium profile predicted by pseudo-first-order Langmuir adsorption kinetics, consistent with a largely irreversible mode of aptamer–bacteria binding within the timescale of the assay. Furthermore, baseline stability was confirmed by maintaining the aptamer-functionalized sensor output within 0.7–1.2 mV over 180 min (Section 3.1). In addition, analysis of the time-course response data (Supplementary Material Table S3) showed that, after reaching saturation, the sensor signal exhibited only limited fluctuations without a systematic monotonic drift, demonstrating good short-term stability. The differential sensing/reference architecture further minimizes common-mode drift caused by temperature, supply voltage variation, and reference circuit instability, thereby improving measurement reliability. Long-term storage drift was not investigated in the present study and will be addressed in future work.

For whole-cell bacterial analytes, the fitted time constant (*τ*) should be interpreted as an apparent response time describing the overall approach to equilibrium, which includes contributions from bacterial transport to the sensing surface and aptamer-mediated capture, rather than as a direct measure of the intrinsic aptamer–bacteria binding kinetics. Nonlinear regression of each response curve to a single-exponential model, *V_Out2_*(*t*) = *V_max_*(1 − e^−t/τ^), where *V_max_* denotes the equilibrium output voltage and *τ* is the apparent response time constant, equal to the inverse of the pseudo-first-order association rate constant, *k_obs_* = 1/*τ*, yielded *R*^2^ > 0.85 (set by HAS-87 at 0.852) for all six analytes (Table 4), confirming that a single rate-limiting adsorption process adequately describes the approach to equilibrium for the target pathogen and genuine clinical/environmental analytes. Fitted τ values ranged from a minimum of 9.8 min (TS-E4) to ~22.7 min for the test sample with 87 CFU/mL analytes.

Because *k_obs_* = *k_on_* × *C* + *k_off_*, comparing the two calibration concentrations (TS-E2, 10^2^ CFU/mL, *k_obs_* = 0.062 min^−1^; TS-E4, 10^4^ CFU/mL, *k_obs_* = 0.054 min^−1^) provides a direct check on whether binding is reaction-limited or diffusion-limited within this platform. A purely reaction-limited process would predict an approximately 100-fold increase in *k_obs_* across this 100-fold concentration range; instead, *k_obs_* was essentially unchanged or, if anything, marginally lower at the higher concentration. This concentration independence indicates that, within the range and pad geometry tested here, the rate-limiting step is diffusive transport of intact bacterial cells to the sensing surface rather than the intrinsic aptamer–LPS binding reaction, consistent with the small sensing pad footprint (69 µm × 57 µm) and the comparatively slow diffusivity of whole bacterial cells relative to small-molecule analytes typically used to validate reaction-limited Langmuir kinetics.

It is important to distinguish two physically distinct effects that jointly shape the observed response. Diffusion-limited mass transport, indicated by the concentration-independence of *k_obs_*, governs the rate at which the signal approaches equilibrium; it does not by itself determine the equilibrium signal ceiling. The mild sub-linearity observed at the highest tested concentration (Section 3.4) instead reflects a separate, equilibrium-level effect: progressive depletion of available aptamer binding sites, as predicted by the Langmuir adsorption framework. These two effects are not in tension; a diffusion-limited approach to a finite-capacity equilibrium is the expected behavior of a surface-based affinity sensor operating near its upper working range. Separately, the largely irreversible character of binding inferred in this study rests on the absence of measurable signal decay during the post-wash rest period (Section 2.6), not on the concentration-independence of *k_obs_*, which instead supports the diffusion-limitation conclusion above; we distinguish these as two independent lines of evidence rather than a single argument.

Mechanistically, the Langmuir framework assumes a finite population of energetically equivalent, non-interacting binding sites on the gold sensing pad, each capable of engaging a single bacterial cell (or LPS epitope), with adsorption proceeding until the available sites approach saturation. The close fit to this model across chemically and biologically distinct analytes, calibration standards (TS-E2, TS-E4), a genuine clinical specimen (PS-4.27E3), and a hospital-acquired environmental specimen (HAS-87) indicates that the underlying adsorption mechanism is conserved across sample matrices. The two non-target *E. coli* strains, however, diverge from this picture in two compounding ways: at the same 10^4^ CFU/mL concentration, K-12 reached only ~23% of the target pathogen’s equilibrium signal (45.7 mV vs. 196.0 mV for TS-E4), which is modestly above the 14–22% relative cross-reactivity reported in Table 3 (likely reflecting the wide fitting uncertainty on K-12’s *V_max_*, ±24.5 mV), while its apparent *τ* (~70 min) was roughly 3.8-fold longer than the target’s, albeit also with substantial fitting uncertainty. ETEC-E4’s response remained below 22 mV throughout the observation window without clearly approaching equilibrium at all, precluding a reliable *V_max_*/*τ* estimate. Together, the appreciably slower apparent kinetics and lower equilibrium amplitude of the non-target strains show that the aptamer’s O157:H7 selectivity manifests as a combined rate-and-affinity effect rather than a purely thermodynamic, equilibrium-only discrimination.

The markedly slower apparent kinetics of the non-target strains at the same nominal concentration (10^4^ CFU/mL) reflect a shift in the rate-limiting step rather than a contradiction of the diffusion-limitation argument above. For the high-affinity target pathogen, aptamer capture upon surface contact is fast and largely irreversible, so the observable approach to equilibrium is governed by mass transport of cells to the sensing surface (diffusion-limited regime). For the lower-affinity non-target strains, capture upon contact is comparatively slow and/or more readily reversible, so the observable rate-limiting step shifts to the aptamer–LPS binding reaction itself (reaction-/affinity-limited regime), producing the substantially longer apparent ***τ*** observed for K-12 and the non-plateauing response observed for ETEC. This dual-regime picture (diffusion-limited kinetics for a high-affinity target and reaction-limited kinetics for low-affinity non-targets) directly explains, rather than conflicts with, the selectivity results of Section 3.6, and is consistent with kinetic behavior widely reported for other surface-based affinity biosensors distinguishing high- from low-affinity analytes.

From an applied standpoint, a saturation time of ~10–23 min for the target pathogen (with full practical plateau by ~30–32 min) is well matched to point-of-care requirements: it is short enough to support same-visit clinical decision-making, yet long enough to permit a reliable, operator-independent fixed-endpoint readout rather than requiring precisely timed kinetic measurements. Combined with the platform’s microwatt-level power consumption and monolithic amplification, this response time supports a simple, fixed-endpoint measurement protocol for future point-of-care implementations, without the need for continuous real-time monitoring hardware.

## 4. Discussion

This study demonstrates that a fully monolithic Bio-CMOS aptasensor can detect intact *Escherichia coli* O157:H7 cells directly in patient-derived and hospital-acquired specimens, with an analytical LOD (~3.74 CFU/mL) and empirical clinical-matrix LOD (~11 CFU/mL) that compare favorably with previously reported aptamer- and antibody-based biosensors for this pathogen. Colorimetric aptasensors using polydiacetylene vesicles conjugated to a truncated LPS-binding aptamer have reported detection ranges of 10^4^–10^8^ CFU/mL, with specificity confirmed by microscopy, roughly four to five orders of magnitude less sensitive than the present platform [14]. Electrochemical microelectrode aptasensors targeting LPS have achieved detection as low as 267 cells/mL within 30 s, but without demonstration in an environmental or clinical sample, precisely the validation gaps this study addresses [32]. More recent nanocomposite-enhanced electrochemical aptasensors combining gold nanoparticles with reduced graphene oxide have been developed to increase surface area and aptamer loading on a glassy carbon electrode for *E. coli* O157:H7 detection in real samples, illustrating that nanomaterial enhancement and CMOS monolithic integration represent two complementary, non-mutually exclusive strategies for improving aptasensor performance; combining the two is a logical next step for this platform.

The reproducibility observed across the three parallel sensing pads (inter-pad *SD* < 1.5 mV) is notable in the context of the wider CMOS biosensor literature, where multi-channel reproducibility is inconsistently reported. Prior CMOS-integrated bacterial biosensors have largely relied on bacteriocin-based potassium-selective ISFET arrays that identify bacteria by measuring potassium efflux following bacteriocin-induced pore formation in the cell envelope, using a CMOS chip connected to an external computer for signal processing. This is a fundamentally different, indirect transduction mechanism relative to the aptamer–LPS binding measured here, and one that, like most reported CMOS platforms, has not been validated in complex clinical matrices [33]. Similarly, extended-gate FET architectures, conceptually related to the reference/sensing pad separation used in the present design, have been applied to label-free whole-cell bacterial detection as an alternative to slower, more resource-intensive PCR-based methods, reinforcing that separating the sensing surface from the transistor channel is an increasingly favored strategy for protecting sensitive electronics from direct liquid exposure while retaining label-free operation. This design philosophy is consistent with recent efforts toward self-calibrated, external-reference-free capacitive/electrochemical readout front-ends for biosensing [29], and with monolithic lab-on-CMOS platforms integrating sensing and signal conditioning on a single die for other bacterial-monitoring applications [30].

The present platform’s engineering specifications provide a concrete basis for reasoning about its translational path. Fabrication in a standard 65 nm TSMC process, rather than a specialized or custom biosensor process, is directly compatible with existing multi-project-wafer and dedicated foundry runs, supporting wafer-level manufacturability and volume-scalable, low-per-unit-cost production without requiring a bespoke fabrication line. The fully on-chip analog front-end (~100 V/V gain, 101.5 μW total static power at a 1 V supply) already eliminates the external reference electrodes and off-chip amplification that constrain the portability of many reported CMOS biosensors, and its microwatt-level power budget is compatible with coin-cell or energy-harvested operation, an important prerequisite for a handheld or disposable-cartridge point-of-care instrument. Full field deployment will additionally require the integration of on-chip or companion analog-to-digital conversion and a low-power wireless or USB digital interface to replace the bench PicoLog data logger used here, packaging beyond the DIP-40 bench carrier into a chip-scale or flip-chip format compatible with a disposable, single-use microfluidic cartridge, and characterization of chip and reagent stability over realistic storage and shelf-life conditions. Multiplexing represents a further natural extension: the current three-pad architecture, presently configured as redundant channels of a single aptamer to establish inter-pad reproducibility (Section 3.4), could be repurposed with minimal redesign to house distinct aptamers per channel, enabling simultaneous multi-pathogen detection on the same monolithic die. None of these steps requires new bench experiments to describe a roadmap, but each represents a distinct engineering validation study in its own right and is identified explicitly as future work.

The modest matrix-induced signal deviation observed between buffer calibration standards and patient-derived specimens (~5.7% at matched concentrations) is smaller than deviations typically reported for PCR-based methods, which remain susceptible to inhibition by components of complex clinical matrices, supporting the manuscript’s framing that whole-cell, label-free electrical transduction may be comparatively robust to matrix interference relative to nucleic acid amplification chemistry, though a head-to-head comparison against PCR on the same specimen set was outside this study’s scope and would be a valuable follow-up. The selectivity data, showing strong discrimination of *E. coli* O157:H7 from six related and unrelated bacterial species, with the highest (but still modest, 12–22%) cross-reactivity confined to other *E. coli* pathotypes sharing overlapping LPS epitopes, are consistent with the aptamer’s originally reported LPS-directed recognition mechanism and are in line with cross-reactivity patterns described for other LPS-targeting aptasensors in the broader literature on aptamer-based pathogen biosensors.

The rapid, ~32 min approach to signal saturation observed across all analytes tested is an important practical attribute of the platform, since it places the assay’s total turnaround time, including the 40–60 min incubation window used in the present protocol, well within the range required for same-visit, point-of-care decision-making, in contrast to the hours-to-days turnaround of culture-based confirmation. The close agreement between the observed response curves and pseudo-first-order Langmuir adsorption kinetics further supports the interpretation that aptamer–bacteria binding at the sensing surface is dominated by a single, largely irreversible adsorption process rather than multiple competing binding modes, consistent with the high selectivity and low baseline drift reported in Section 3.1 and Section 3.6. The near-concentration-independence of *k_obs_* (Section 3.7) further indicates that analyte transport, rather than intrinsic aptamer affinity, sets the pace of signal development at this pad geometry, a distinction relevant to further miniaturization, since shrinking the sensing pad could paradoxically slow rather than speed the response.

Several limitations should be acknowledged. This study was designed as a pilot, proof-of-concept clinical feasibility evaluation rather than a diagnostic-accuracy study, and its conclusions should be interpreted accordingly. Diagnostic sensitivity, specificity, positive predictive value, and negative predictive value are not reported here, since the modest cohort size (10 PS, six HAS specimens) cannot support confidence intervals of acceptable precision for these metrics; a prospectively enrolled, multi-site cohort of substantially larger size, ideally following a STARD-compliant diagnostic-accuracy design, will be required before formal clinical-accuracy claims can be made. First, the clinical validation set, while a genuine strength relative to prior buffer-only studies, remains modest in size (10 PS and six HAS specimens); larger, multi-site cohorts will be needed to establish clinical sensitivity/specificity with confidence intervals suitable for diagnostic-accuracy reporting. Second, the platform currently reports a single analyte per chip design (three redundant channels of the same aptamer) rather than true multiplexed, multi-pathogen detection; extending the sensing pad array to house distinct aptamers per channel is a natural next step and is consistent with the multi-pathogen ambition stated in the Introduction. Third, while the on-chip analog front-end removes the need for external reference electrodes, itself a meaningful step toward field deployability, full point-of-care translation will additionally require on-chip or embedded digital readout, sample-loading automation, and stability data over realistic storage and shelf-life conditions, none of which were characterized here. Fourth, the calibration curve used to derive sensitivity and the LOD has now been re-analyzed using a four-parameter logistic model (Section 3.3), which substantially improves the goodness of fit relative to the original linear approximation and removes the systematic residual structure associated with the linear fit; the linear model is retained only as a practical local approximation within the mid-range of the dynamic range. The nonlinear fit’s lower-asymptote and slope-factor parameters nonetheless carry wider uncertainty than its inflection-point and upper-asymptote parameters at the current calibration density (five non-zero concentration levels), and additional calibration points would further refine these estimates in future work. Fifth, the selectivity panel, while spanning a phylogenetic gradient from closely related *E. coli* pathotypes to Gram-positive organisms lacking LPS entirely, did not include other Shiga-toxin-producing *E. coli* serogroups (e.g., O104:H4, O26, O111) that share the Shiga-toxin pathotype with O157:H7 but present distinct O-antigen structures; distinguishing the platform’s response to these near-neighbor STEC serogroups is an important and currently unaddressed question that will require testing additional strains not available in the present study. Sixth, the present protocol standardizes ionic strength via a dilution step and was not evaluated on fully undiluted, minimally processed native specimens; while this represents a genuine boundary of the current analytical validation, it is a tractable engineering target for future work, for example, through porous or permeable surface coatings that extend the effective screening length at the sensing interface, high-frequency AC interrogation strategies, or on-chip/microfluidic sample-preparation modules integrated upstream of the sensing pads, each of which has precedent in the FET biosensor literature for operation at or near full physiological ionic strength.

Despite these limitations, the practical significance of this work lies in its combination of microwatt-level power consumption, a fully monolithic sensing-plus-amplification architecture requiring no external reference electrode, and—most importantly—validated performance in real patient-derived and hospital-acquired specimens rather than buffer alone. This combination has, to our knowledge, not been previously demonstrated together in a single CMOS aptasensor platform for *E. coli* O157:H7, and it directly addresses the translational gap identified in point-of-care biosensor reviews, which consistently note that aptasensor design for infectious-disease point-of-care testing must bridge laboratory-validated analytical performance and real-world clinical deployment. The present results provide a practical, quantitatively characterized foundation for that bridge and for the future multi-pathogen diagnostic arrays proposed in the Introduction.

## 5. Conclusions

This study demonstrates a monolithically integrated, label-free Bio-CMOS aptasensor capable of detecting *Escherichia coli* O157:H7 directly in patient-derived and hospital-acquired specimens, without enrichment, labeling, or external reference electrodes. The platform achieved an analytical limit of detection of ~3.74 CFU/mL and an empirical clinical-matrix limit of detection of ~11 CFU/mL, with only modest matrix-induced signal deviation relative to buffer standards and strong selectivity against six related and unrelated bacterial species. Three independently addressable sensing pads showed high inter-pad reproducibility, supporting the robustness of the differential sensing architecture. Kinetic analysis further showed that sensor responses closely followed pseudo-first-order Langmuir adsorption behavior, reaching saturation within approximately 16–25 min for the target pathogen, with slower kinetics for non-target strains, a response time well suited to same-visit clinical decision-making. Operating at 101.5 µW total power with an on-chip ~100 V/V analog front-end, the platform’s compact, self-contained design represents a practical step toward field-deployable, point-of-care pathogen diagnostics. Its principal current limitations are a clinical validation cohort of modest size and a single-analyte (rather than multiplexed) sensing configuration; addressing both will be necessary before the platform can be considered for prospective clinical-accuracy studies or extended to simultaneous multi-pathogen detection. With these extensions, the architecture described here offers a scalable foundation for compact, low-power, multi-pathogen diagnostic arrays suited to decentralized and emergency healthcare settings. Realizing this potential will require a prospectively enrolled, adequately powered diagnostic-accuracy study, conducted according to standard diagnostic-accuracy reporting guidelines, as the necessary next step beyond the present feasibility evaluation.

## Figures and Tables

**Table 1:** Biochip specifications.

Parameter	Value
Technology	65nm TSMC
Chip area	1.5 mm × 0.7 mm
Sensing area	69 µm × 57 µm
Power supply	1 V
Power consumption	AMP0, AMP1, *I_Bias_* block: 69.84 µA × 1 V
	BVR Block: 31.66 µA × 1 V
	Total: 101.5 µA × 1 V
*V_ref_* fluctuation	4.41%
Gain	100 (40 dB)
Bandwidth	50 kHz

**Table 2:** Electrical response of the aptasensor for calibration standards (TS series). Aptamer + MCH represents the blank (analyte-free) sample.

Sample (CFU/mL)	Output Signal (mV)		
	Out2_1	Out2_2	Out2_3
Blank	1.05	1.35	1.17
1	2.11	1.76	1.33
10	17.22	18.45	18.05
100	52.65	53.05	53.21
1000	108.53	109.34	110.23
10,000	179.54	180.22	182.61

**Table 3:** Cross-reactivity to enterobacteriaceae. Selectivity of the Bio-CMOS aptasensor against six bacterial species at 10^2^ and 10^4^ CFU/mL.

Organism Sample CFU/mL		Out2 (mV)		Relative Signal (%)
		10^2^	10^4^	
G. Negative	*E. coli* O157:H7 (*TS*)	~53	108–110	100%
	*E. coli* K-12	8.1–9.2	16–22	14–22%
	ETEC	7.2–9.0	14–21	12–20%
	*Salmonella typhimurium*	4.3–7.4	9.8–15	8–15%
	*Klebsiella pneumoniae*	3.2–5.2	5.4–11	5–10%
G. Positive	*Staphylococcus aureus*	1.1–3.7	2.3–7.5	2–7%
	*Enterococcus faecalis*	1.0–1.9	2.3–4.9	2–4%

**Table 4:** Fitted kinetic parameters (*V_max_/τ*) and goodness of fit (R^2^) obtained from nonlinear regression of Table 4 time-course data to the single-exponential adsorption model, *V_Out2_*(*t*) = *V_max_*(1 − e^−*t/τ*^), for each of the six analytes tested; *k_obs_* = 1/*τ*.

Analyte	Concentration	*V_max_* (mV)	τ (min)	*k_obs_* (min^−1^)	*R* ^2^
TS-E2	10^2^ CFU/mL	55.7 ± 3.3	16.1 ± 2.1	0.062	0.967
TS-E4	10^4^ CFU/mL	196.0 ± 4.2	18.5 ± 2.2	0.055	0.974
PS-4.27E3	4270 CFU/mL	135.7 ± 4.5	16.0 ± 2.3	0.063	0.959
HAS-87	87 CFU/mL	48.3 ± 5.6	24.5 ± 3.1	0.040	0.852
K-12	10^4^ CFU/mL	45.7 ± 2.8	70.5 ± 6.3 §	0.014	0.890
ETEC	10^4^ CFU/mL	Not Resolvable ^†^	Not Resolvable ^†^	<0.01	0.911

^†^ ETEC’s curve never clearly plateaus in 60 min, so *V_max_/τ* trade-off against each other and cannot be pinned to a precise value (huge SEs if you force the fit: *V_max_* ≈ 375 ± 3255, *τ* ≈ 1000 ± 8880, which is the model telling you it is unidentifiable, not a real estimate). Report *R*^2^ but flag *V_max_/τ* as indeterminate for this analyte specifically. § Note K-12’s fit also carries wide uncertainty (± roughly half the value itself), which is honest and worth stating in-text rather than hiding; it still converges to a finite number, unlike ETEC, but should not be over-interpreted as a precise point estimate either.

## Data Availability

The original contributions presented in this study are included in the article/Supplementary Materials. Further inquiries can be directed to the corresponding authors.

## Supplementary Materials

The following supporting information can be downloaded at: https://www.mdpi.com/article/10.3390/bioengineering13080858/s1. Figure S1. Measured frequency response of the biochip amplifier chain. The system exhibits a flat gain of approximately 40 dB from low frequencies up to 50 kHz, with no observable peaking, indicating stable compensation and adequate phase margin. Figure S2. The complete architecture of one sensing block: (a) bandgap and current biasing generation, (b) high-level block diagram with reference and sensing pads, and (c) transistor-level detail of the differential input and compensation elements. Figure S3. (a) Combined low-voltage bandgap voltage reference (**right**) and regulator (**left**) generating stable *Vbg* ≈ 500 mV, (b) wide-swing current biasing block producing mirrored currents I1, I2, I3 and PMOS gate bias INP1 for the operational amplifier stages. Figure S4. The detailed design of OpAmp1. Figure S5. Bar chart of mean Out2 (mV) vs. sample category (TS-E0 through TS-E4), control was added for comparison, spanning approximately four orders of magnitude in CFU/mL, with error bars (± SD). Table S1. Component features for BVR block. Table S2. Component features for current biasing block. Table S3. Raw time-course response signals (*V_Out2_* in mV) of the Bio-CMOS aptasensor during 60-minute incubation with six analytes: the target pathogen at two calibration concentrations (TS-E2, 10^2^ CFU/mL; TS-E4, 10^4^ CFU/mL), a genuine patient-derived specimen (PS-4.27E3, 4270 CFU/mL), a hospital-acquired environmental specimen (HAS-87, 87 CFU/mL), and two non-target E. coli strains at 10^4^ CFU/mL (K12-E4, ETEC-E4). These data underlie the non-linear regression fits reported in Table 4 and Figure 8.

## Abbreviations

CMOS	Complementary metal-oxide-semiconductor
STEC	Shiga-toxin-producing *Escherichia coli*
HAI	Hospital-acquired infection
HUS	Hemolytic uremic syndrome
PCR	Polymerase chain reaction
ELISA	Enzyme-linked immunosorbent assay
SELEX	Systematic evolution of ligands by exponential enrichment
LPS	Lipopolysaccharide
ISFET	Ion-sensitive field-effect transistor
EG-FET	Extended-gate field-effect transistor
SAM	Self-assembled monolayer
MCH	6-Mercapto-1-hexanol
PBS	Phosphate-buffered saline
TE (buffer)	Tris-EDTA (buffer)
PDMS	Polydimethylsiloxane
TSMC	Taiwan Semiconductor Manufacturing Company
LOD	Limit of detection
CFU	Colony-forming unit
SD	Standard deviation
TS	Test (calibration) sample
PS	Patient-derived specimen
HAS	Hospital-acquired (environmental) specimen
BVR	Bandgap voltage reference
ATCC	American Type Culture Collection
ETEC	Enterotoxigenic *Escherichia coli*
ssDNA	Single-stranded DNA
HPLC	High-performance liquid chromatography
5-FAM	5-Carboxyfluorescein
DIP	Dual in-line package
IUPAC	International Union of Pure and Applied Chemistry
