# Supplementary material for: A Monolithic, Thiol-Functionalized Au-Based Bio-CMOS Aptasensor for Rapid, Label-Free Detection of Escherichia coli O157:H7 in Patient-Derived and Hospital-Acquired Specimens
Source: Bioengineering (Basel). 2026 Jul 25;13(8):858. doi: 10.3390/bioengineering13080858 (PMC13509967; doi:10.3390/bioengineering13080858)

Supplementary Material S1

BioCMOS Design and Architecture

The design incorporates separate supply domains for the reference block and the operational amplifiers with current-biasing circuits, enabling precise measurement of individual power contributions. At a nominal supply voltage of 1 V, OpAmp0, OpAmp1, and the biasing blocks consume 69.84 μA, while the reference block requires 31.66 μA, yielding a total static power of 101.5 μW (as indicates in Table 1), suitable for portable biosensing platforms.

The on-chip reference circuit exhibits excellent supply and temperature stability, with an output variation of 0.088 mV/V across 0.7–1.2 V and a maximum shift of 8.47 mV between 10 and 80 °C. The complete amplifier chain shows a flat gain of ~40 dB (Av ≈ 100) up to ~50 kHz without peaking (Supplementary Material Figure S1), indicating adequate phase margin and robust closed-loop behavior under varying sensor loading conditions. The input-referred noise integrated from 0.1 Hz to 50 kHz is 16.6 μVrms, corresponding to a signal-to-noise ratio of 48 dB for a 2 mV test signal. Taking three times the integrated noise as a conservative detection threshold (~50 μV at the sensing node) and using the measured gain (Av≈100), the minimum resolvable surface-potential change is on the order of 50 μV at the pad, which appears as ~5 mV at Out2.

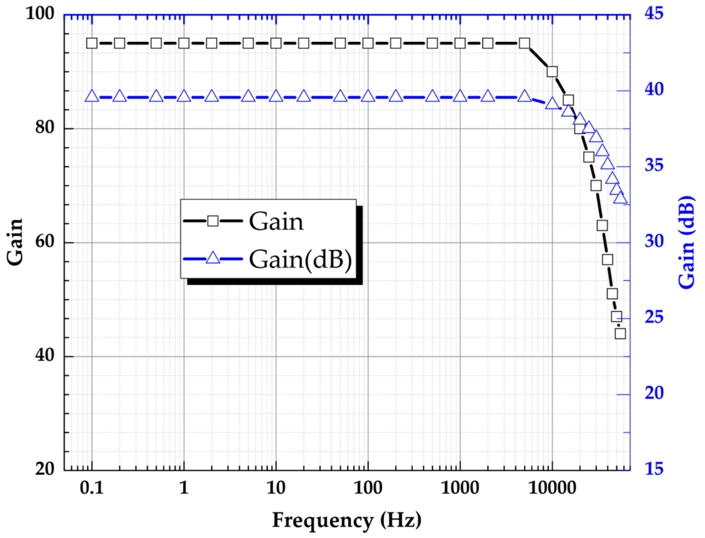

Supplementary Material Figure S1. Measured frequency response of the biochip amplifier chain. The system exhibits a flat gain of approximately 40 dB from low

frequencies up to 50 kHz, with no observable peaking, indicating stable compensation and adequate phase margin.

Biochip Readout Circuit

The sensing blocks employ an extended-gate field-effect transistor (EG-FET) configuration implemented within a standard CMOS process. Upon specific binding, conformational changes and charge redistribution occur at the electrode–electrolyte interface, inducing a shift in local surface potential (typically a few millivolts). The gold pad is connected through top-metal vias to the extended gate of the PMOS transistor MP0 in AMP0 (Supplementary Material Figure S2c). This configuration physically isolates the chemically sensitive electrode from the MOSFET channel, enhancing robustness against liquid-induced degradation, process variations, and environmental noise while maintaining high sensitivity. The sensing voltage Vin at MP0’s gate is differentially compared with the reference voltage *Vref* from an identically fabricated reference pad that is cleaned and passivated but not functionalized with the E. coli-specific aptamer, thereby providing a chemically stable, non-responsive baseline. This differential approach suppresses common-mode disturbances such as pH drift, ionic strength fluctuations, and temperature variations, conditions frequently encountered in complex hospital-acquired samples.

A reference block (Supplementary Material Figure S2a) generates a temperature-compensated voltage *Vbg*, which biases the PMOS current sources in the differential stages. A current-biasing block produces three matched currents (I1, I2, I3) using wide-swing current mirrors, minimizing static power consumption and silicon area, critical for scalable multi-channel biosensor arrays.

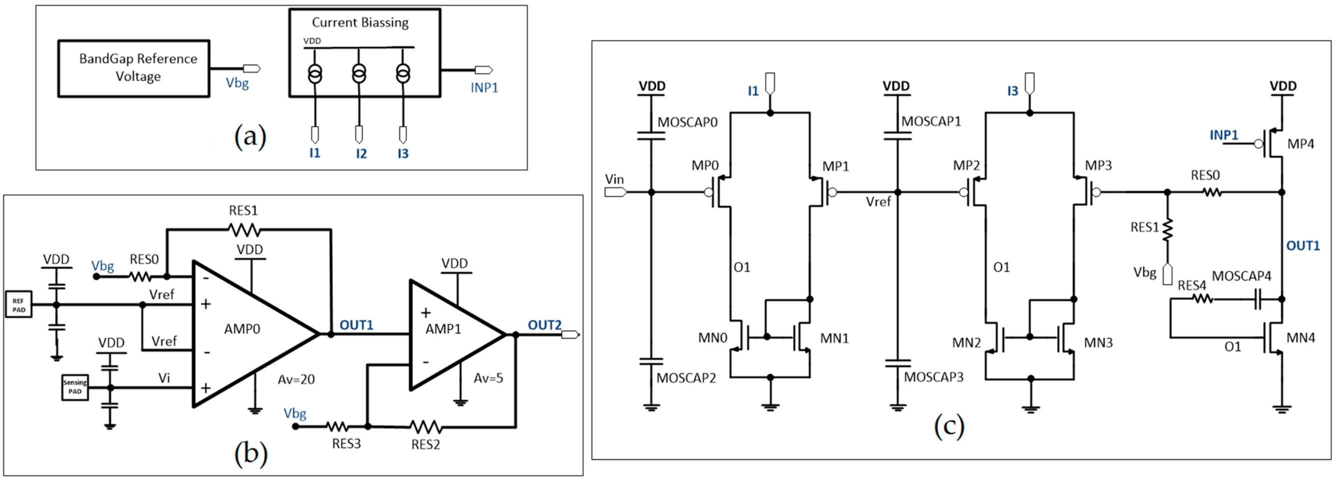

Supplementary Material Figure S2. The complete architecture of one sensing block: (a) bandgap and current biasing generation, (b) high-level block diagram with reference and

sensing pads, and (c) transistor-level detail of the differential input and compensation elements.

Illustrated in Supplementary Material Figure S2b, AMP0 employs a PMOS differential input pair (MP0/MP1) with NMOS active loads (MN0/MN1), providing a nominal gain of ~20. AMP1, a common-source amplifier with PMOS active load, adds an additional gain of ~5, producing an overall amplification suitable for downstream digitization or external interfacing. The output (Out2) is routed to an I/O pad for further processing. Negative-feedback networks (RES0–RES3) stabilize the operating points, define closed-loop gains, and ensure linearity across the expected signal range. MOS capacitors (32 μm × 32 μm, ≈2.1 pF) act as charge integrators and AC-coupling elements, preserving transient charge variations from the sensing pad and preventing rapid leakage, crucial for detecting weak biosensing signals.

BVR and Current Biasing

Supplementary Material Figure S2 illustrates the on-chip reference and biasing circuits that ensure stable, low-power operation of the Bio-CMOS readout system. The Bio-CMOS chip employs a MOSFET-based subthreshold voltage reference (Supplementary Material Figure S3a, **right**) that provides a bandgap-like, temperature-compensated bias without using bipolar devices. In contrast to a classical 1.2 V bandgap reference, the implemented circuit generates a lower reference level of approximately *Vref* ≈ 500 mV, which is well suited to the 1 V supply used in this work. The reference core combines CTAT and PTAT components derived from MOS transistors operating in weak inversion, yielding a temperature-stable output over the 10–80 °C range. The compact PMOS-based voltage regulator (Supplementary Material Figure S3a, **left**) uses resistive feedback to generate the final reference voltage *Vref* ≈ 500 mV, providing a clean, supply-independent bias for the sensitive analog stages.

This subthreshold reference feeds a set of current-biasing branches that establish the operating points of the on-chip amplifiers and sensing front-end (Supplementary Material Figure S3b). The generated bias currents are mirrored into the differential input stages and gain stages of AMP0 and AMP1, ensuring consistent transconductance and gain across process and temperature variations. As explained above, the reference block itself consumes 31.66 μA at 1 V, while the op-amp and biasing circuitry draw 69.84 μA, resulting in a total static power of 101.5 μW. Supplementary Material Tables S1 and S2 summarize the key performance properties of the reference and current-biasing components.

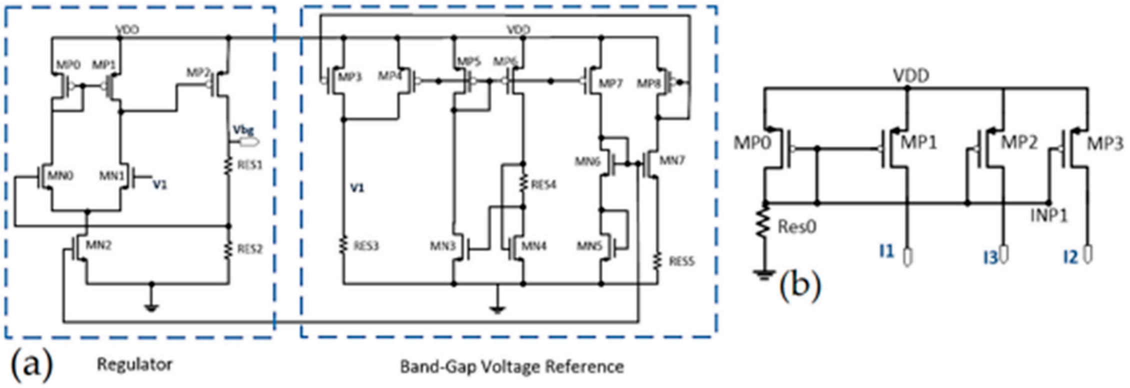

Supplementary Material Figure S3 (a) Combined low-voltage bandgap voltage reference (**right**) and regulator (**left**) generating stable $V_{bg} \approx 500$ mV, (b) wide-swing current biasing block producing mirrored currents I1, I2, I3 and PMOS gate bias INP1 for the operational amplifier stages.

Supplementary Material Table S1. Component features for BVR block.

Component	Parameter	Component	Parameter
MN0	10µm/1µm	MP0	10µm/4µm
MN1	10µm/1µm	MP1	10µm/4µm
MN2	10µm/1µm	MP2	10µm/1µm
MN3	8µm/1µm	MP3	10µm/1µm
MN4	8µm/1µm	MP4	6µm/1µm
MN5	80µm/500nm	MP5	10µm/1µm
MN6	40µm/5µm	MP6	20µm/1µm
MN7	80µm/5µm	MP7	10µm/1µm
RES1	345.121 kΩ	MP8	60µm/130nm
RES2	299.974 kΩ	RES4	22.0043 kΩ
RES3	400.8 kΩ	RES5	115.04 kΩ

Supplementary Material Table S2. Component features for current biasing block.

Component	Parameter	Component	Parameter
MP0	10µm/2µm	MP3	20µm/130nm
MP1	10µm/130nm	RES0	300 kΩ
MP2	10µm/130nm		

OPAMPS

The readout circuitry employs a cascoded multi-stage amplification scheme to achieve high sensitivity and robust noise rejection for detecting minute charge variations induced by analyte–aptamer binding. The small differential signal between the sensing pad and the matched reference pad is first processed in OpAmp0, which performs initial subtraction and moderate gain, converting the charge-induced surface-potential shift (typically in the mV range) into a usable voltage at Out1.

This amplified difference is then fed into OpAmp1 (Supplementary Material Figure S4), a classical two-stage amplifier with a differential input stage followed by a common-source gain stage, stabilized by Miller compensation with a series resistor. This topology provides substantial additional gain while suppressing broadband noise, offset errors, and residual common-mode disturbances that could otherwise corrupt the weak biosensing signal. A feedback network defines the closed-loop gain and operating point, while integrated MOS capacitors act as charge-storage elements at the high-impedance input nodes, preserving the charge delivered from the sensing electrode and preventing rapid dissipation through leakage paths. Together, these stages deliver an overall gain of ~40 dB with low noise and stable operation across the intended bandwidth.

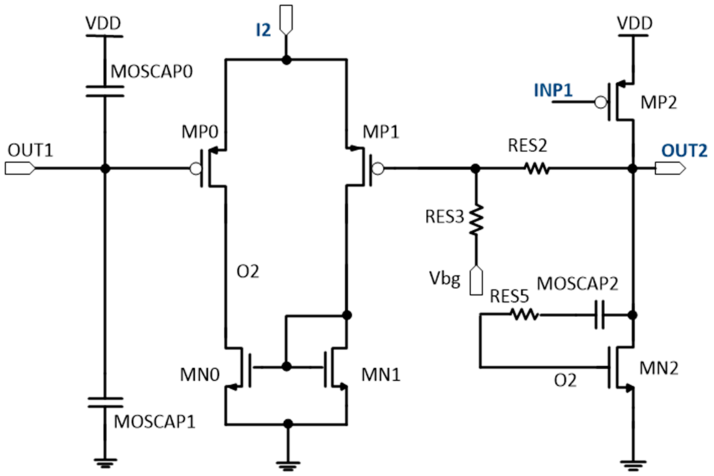

Supplementary Material Figure S4. The detailed design of OpAmp1

Supplementary Material Table S3. Raw time-course response signals (V_{Oou2} in mV) of the Bio-CMOS aptasensor during 60-minute incubation with six analytes: the target pathogen at two calibration concentrations (TS-E2, 10^2 CFU/mL; TS-E4, 10^4 CFU/mL), a genuine patient-derived specimen (PS-4.27E3, 4,270 CFU/mL), a hospital-acquired environmental specimen (HAS-87, 87 CFU/mL), and two non-target E. coli strains at 10^4 CFU/mL (K12-E4, ETEC-E4). These data underlie the non-linear regression fits reported in Table 4 and Figure 8.

Time minutes	TS-E2 mV	TS-E4 mV	PS-4.27E3 mV	HAS-87 mV	K12-E4 mV	ETEC-E4 mV
0.1	1.35	2.45	1.45	1.3	1.15	1.2
5	9.37	36.7	19.4	4.9	1.73	1.4
10	23.2	78.3	65.8	12.8	2.5	2.1
15	34.6	93.6	74.4	14.7	4.3	3.7
20	37.5	121.64	91.3	20.4	8.34	3.6
25	45.34	163.7	114.8	30.1	11.35	6.54
30	53.85	165.4	133.4	47.5	21.2	11.45
35	53.1	180.6	122.8	45.2	20.5	13.2
40	53.25	178.32	130.2	43.4	24.5	16.33
45	52.01	176.4	128.5	42.56	22.4	19.64
50	50.6	179.5	125.45	40.2	24.17	21.8
55	51.3	180.3	122.8	36.7	22.7	20.16
60	52	178.6	126.2	39.4	23.04	18.3

Supplementary Material Figure S5. Bar chart of mean Out2 (mV) vs. sample category (TS-E0 through TS-E4), control was added for comparison, spanning approximately four orders of magnitude in CFU/mL, with error bars (± SD).

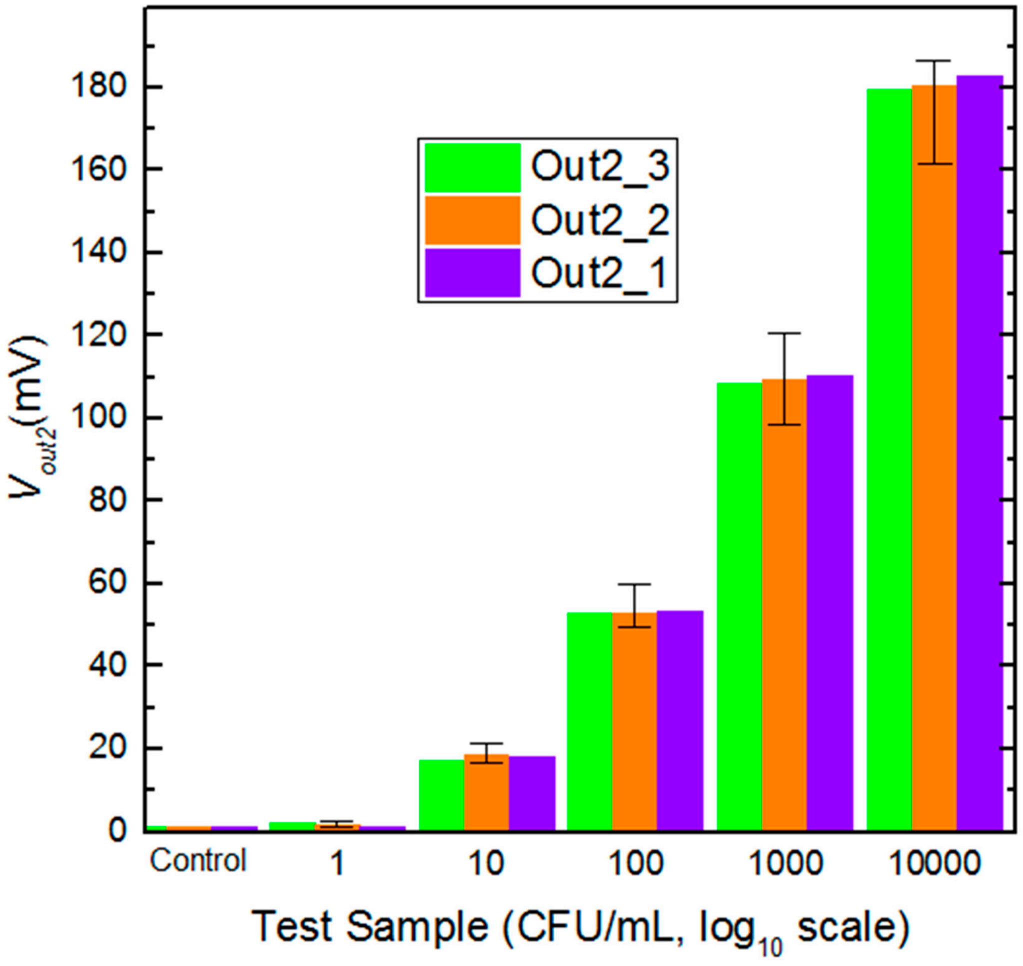

Supplement: Supplementary file 1 [file bioengineering-13-00858-s001.zip › Supplementary_S1_BioCMOS_Design_Architecture.pdf]
